# Ligas do trauma – the structure of student societies as teaching method of trauma in brazil

**DOI:** 10.1186/1757-7241-23-S2-A24

**Published:** 2015-09-11

**Authors:** Bruna Latronico, L S Souza, Camila O Morais, Ismael K Dornelles, Atila V Velho

**Affiliations:** 1 Liga do Trauma da UFCSPA, Department of Surgery of Universidade Federal de Ciências da Saúde de Porto Alegre, Porto Alegre, Brazil

## Introduction

“*Ligas do Trauma*” (translated as “Trauma Leagues”) are **Student Societies** dedicated to learn Trauma as an extracurricular subject within Medical School. Students ranging years 1 to 6 participate in the society being stimulated to give lectures, write papers and clinical reviews, organize events to educate healthcare workers (reaching nurses, Rescue Team volunteers, Firemen, etc.), and also spread information to lay community. Brazil is a massive country with an equally gigantic incidence of Trauma Injuries, thus the professional must be trained to identify and treat Trauma regardless the future choice of specialization. This structure of societies is spreading with more than 50 “Leagues” installed in different Brazilian Universities.

## Objectives

Using “*Liga do Trauma da UFCSPA* ” as a model, we want to expose our inner structure and discuss the applicability of our projects and interventions.

## Methods

Presentation of the Society. Expose the projects: 1. Present weekly lectures on *First Aid, Pre-Hospital* and *Intra-Hospital Care* to all Health Faculties of UFCSPA; 2. Host biannual courses on *Practical Immobilization in Trauma, Clinical Emergencies, Suture and Surgical Knots,* all open to public; 3. Schedule visits to Primary and High Schools with campaigns and teaching material on *Prevention of Domestic Injuries* and *Basic Life Support* (*BLS*); 4. Join work with “Ligas” in national impact projects, such as the “*National CPR Day* ”; 5. Colaboration with the Pre-hospital Care System in Brazil (SAMU – *Serviço de Atendimento de Urgência*) with active participation on FIFA World Cup 2014, among many others.

## Conclusions

Student Societies are growing in Brazil with similar structures and very effective measures of teaching Trauma. The student is seen as capable of getting involved with the subject before reaching higher Surgical or Emergency levels in the Medical Career. Being part of a “League” can subjectively lead the student to follow a career path in Trauma.

